# Performance Management Counters from Live 5G, 4G and 2G Radio Access Network

**DOI:** 10.1038/s41597-026-07723-0

**Published:** 2026-07-03

**Authors:** Peter Lehoczký, Matúš Turcsány, Laura Krajčovičová, Filip Zatroch, Marcel Kajan, Marek Galinski

**Affiliations:** 1 Faculty of Informatics and Information Technologies, Slovak University of Technology, Bratislava, Slovakia; 2 Ericsson Slovakia, Bratislava, Slovakia

## Abstract

We introduce an open live multi-technology cellular network dataset derived from real, operational base stations of a commercial mobile operator in Slovakia. It provides a rare look inside live 2G, 4G and 5G Radio Access Networks (RANs) through Performance Management (PM) counters collected at 15-minute intervals and mapped to cell-level granularity. Each baseband processes multiple radio configurations, enabling multi-band and multi-generation correlation studies across real network deployments. Unlike any previously released resource, this dataset exposes real energy consumption values alongside radio and user-level performance indicators such as utilisation, Channel Quality Indicator (CQI), number of Radio Resource Control (RRC) connected and active users, uplink and downlink data volume and Multiple-Input Multiple-Output (MIMO) rank for all network cells where applicable. It supports research in energy-aware RAN optimisation, data-driven network management and cross-layer performance modelling. The dataset has been validated for internal consistency and is released under the Creative Commons Attribution (CC BY) license to foster reproducible and open telecommunications research.

## Background & Summary

The availability of real-world mobile network performance data is extremely limited due to confidentiality, security and operational constraints. Yet, such data are essential for understanding and optimising Radio Access Networks (RANs), particularly in the transition from 4G to 5G technologies and future 5G to 6G migration. As mobile traffic and energy demands continue to increase, network operators face the challenge of maintaining throughput efficiency while minimising energy consumption. This dataset offers a rare and unique opportunity to investigate these relationships on commercial infrastructure and to build predictive models grounded in live network behaviour.

Modern RANs generate vast amounts of telemetry through Performance Management (PM) counters, which capture network behavior across different network elements, bands and technologies. These counters form a critical foundation for data-driven network management. However, datasets based on real operational networks are rarely available to the research community. The lack of public datasets has been widely acknowledged. For instance, Almeida *et al*. note the difficulty in advancing 5G analysis due to limited data. They were among the first to analyze commercial 5G data from a live network, examining 27 weeks of hourly telemetry (134 metrics per site) from 13 base stations in a Portuguese city center. Their work demonstrated the insights possible with real 5G data, but the dataset itself remained proprietary and was not shared publicly as it contains sensitive business information (the operator’s privacy concerns even prevented showing the cell locations)^1^. Similarly, Eller *et al*. combined base-station performance counters with crowdsourced user measurements to develop a differentiable throughput model for load-aware optimisation. Using approximately 150 cells from an operational Long Term Evolution (LTE) network in Vienna (with Key Performance Indicator (KPI) reports averaged over 15 minutes)^2^, they showed the potential of data-driven parameter tuning through gradient descent.

To our best knowledge, the dataset presented in this work is the first publicly available dataset of its kind in the telecommunications domain. It originates from a live commercial mobile network in Slovakia and captures multi-technology, multi-band base stations providing Global System for Mobile Communications (GSM)/2G, LTE/4G and 5G New Radio (NR) access over extended periods. Each base station in this network follows a modular architecture: a digital baseband unit manages several Remote Radio Units (RRUs) and their sectors. Each radio unit can host multiple carriers and technologies simultaneously, enabling a flexible multi-band configuration per site. The dataset preserves this hierarchy by retaining the mapping between basebands and sectors. By openly providing real network telemetry at a 15-minute granularity across multiple time spans, our contribution unlocks research possibilities previously limited to operators’ internal data.

Our dataset builds on and extends prior measurement collections. A few recent efforts have begun sharing mobile network data. For example, Kousias *et al*. released a multi-technology network measurement dataset spanning 4G, Narrowband Internet of Things (NB-IoT), and 5G Non-Standalone networks^3^. Likewise, Schippers *et al*. introduced the DoNext open dataset, containing two years of 4G/5G mobile network measurements collected through active and passive probing in urban, suburban, and rural environments. DoNext is touted as “the most extensive openly available dataset of 4G and 5G mobile network measurements to date”^4^ and enables machine-learning research on KPIs like throughput and latency. While these resources mark important steps toward open mobile data, they differ fundamentally from our approach. Existing public datasets typically consist of external observations (e.g. user device measurements, drive tests or crowdsourced metrics) or abstracted features, rather than raw internal network telemetry. They are often restricted to specific scenarios or technologies (e.g. either 5G or IoT, but not all at once), and often lack fine temporal resolution for short-term dynamics. In contrast, our dataset exposes the raw sector-level performance counters from a live network with full visibility into a broad set of RAN parameters. It captures three coexisting radio access technologies with real traffic and user behavior at fine time granularity, enabling detailed cross-technology and temporal analyses that were previously infeasible with open data.

While preparing this work, we also examined a recent contribution accompanying the UoMo model, which provides a large-scale collection of mobile network measurements across several 4G and 5G environments. Rather than releasing raw base station parameters, those authors apply a preprocessing pipeline that transforms the original measurements into abstract data tokens – encoded feature bundles without direct mapping to underlying network metrics. Their focus is on learning shared traffic patterns across heterogeneous networks (nine datasets from seven Chinese cities, with additional test sets from China and Germany) to enable model generalisation^5^. While this approach offers valuable insights for universal modelling, the published data do not include interpretable network KPIs, limiting fine-grained analysis or direct operational use. In contrast, our dataset retains full parameter visibility for each sector and time interval, enabling transparent modelling, reproducible experimentation, and detailed investigation of real operational behaviour.

The provided data enable correlation analyses between throughput, traffic load, user density, and energy consumption across multiple time scales. Moreover, it can be used to develop data-driven and Artificial Intelligence (AI)-based models for network performance and energy assessment. The dataset supports systematic feature engineering and selection from a set of physical-layer and user-level KPIs, facilitating predictive modelling of throughput distributions and energy demand, as well as quantitative evaluation of performance degradation under congestion. In addition, it is suitable for applying explainable machine learning methods to identify the most influential factors governing throughput, power consumption or other relevant metrics. Overall, the data provide a foundation for data-driven RAN optimisation, capacity planning, and the evaluation of energy-aware network operation, including investigations of cross-layer dependencies (e.g., joint effects of radio link quality and user density on throughput and power usage).

We anticipate broad reuse of this dataset by researchers in radio network optimisation, machine learning-based performance prediction, energy modeling, and digital twins for cellular networks. By exposing raw sector-level telemetry with fine temporal granularity, the dataset supports both classical statistical analysis and the development of learning-based approaches, all without requiring access to confidential operator systems. It complements existing proprietary studies and simulation-based frameworks by reflecting real operational dynamics - including natural traffic variability, occasional outages, and low-traffic periods - that are hard to emulate.

By providing a transparent and well-documented snapshot of network performance, this dataset contributes to the growing body of open and semi-open telecommunications data resources. It enables cross-domain research on data-driven network management and can serve as a baseline for studies on sustainable, self-optimizing RANs. In line with open-science principles, the data are anonymized and structured to facilitate reproducible research by both academia and industry. Beyond its scientific value, it offers a glimpse into the performance characteristics of mobile infrastructure in Central Europe, and a foundation for future comparative analyses of energy-aware 5G deployments.

To the best of our knowledge, this is the only openly available dataset of this scope that provides raw multi-technology (2G/4G/5G) sector-level PM counters together with real energy consumption measurements from a live commercial network. No prior public dataset offers simultaneously multi-band architecture, fine 15-minute granularity and visibility into infrastructure-side KPIs.

## Methods

### Data collection

The dataset was derived from cellular network data collected at base stations, which serve as fundamental components of the RAN infrastructure. The raw input data were provided by CETIN Networks through a research collaboration and originate from live base stations in a commercial European mobile network. The base stations, in particular the basebands associated with each base station, generate Extensible Markup Language (XML) files in compliance with the 3rd Generation Partnership Project (3GPP) specifications. These files are produced at 15-minute intervals and contain extensive measurements from multiple PM counters at cell level, node level and unit level. These operator-provided records form the source data from which the released dataset was prepared.

**Fig. 1:**
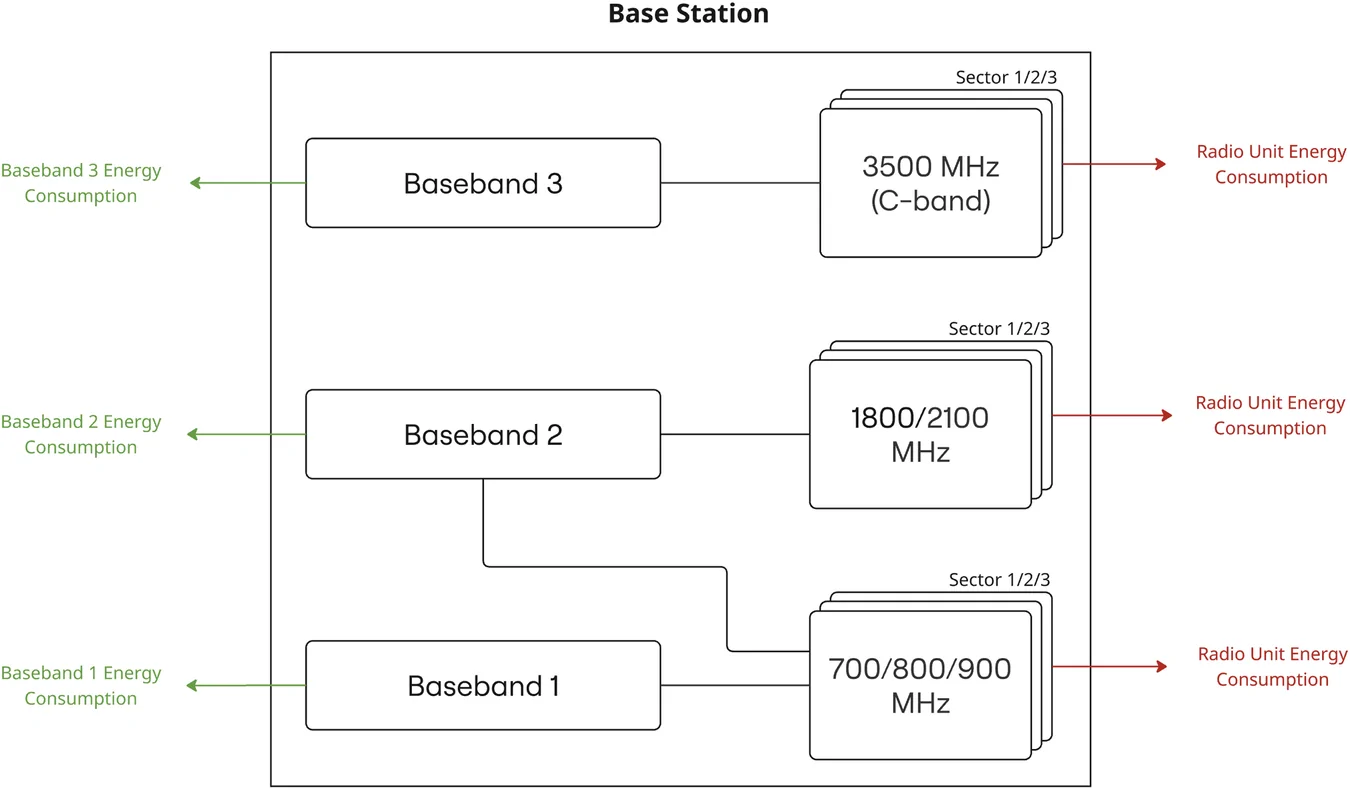
Base station architecture diagram. Solid lines represent physical hardware connectivity.

The base station architecture consists of three baseband processing units and three types of RRUs (Fig. 1). Baseband 1 handles GSM/2G cells and is connected to multi-band RRUs operating in the 700/800/900 Megahertz (MHz) frequencies. Baseband 2 handles all 4G frequency division duplex (FDD) and 5G FDD cells and connects to multi-band RRUs operating at 1800/2100 MHz and 700/800/900 MHz frequencies. Baseband 3 handles 5G time division duplex (TDD) cells and is connected to Massive Multiple-Input Multiple-Output (MIMO) radio units operating in the 3500 MHz C-band spectrum.

Each baseband unit is connected to multiple RRUs configured with multiple cells, creating several sectors (one to three sectors labeled as Sector 1/2/3) in a standard cellular network layout. Energy consumption data is generated at node level, i.e. each radio and each baseband. Therefore, power consumption per cell is not directly observable.

The configured channel bandwidths of the LTE and NR cells included in the dataset are summarised in Table 1. These bandwidths are relevant when interpreting resource block (RB) utilisation values, because the same utilisation percentage corresponds to a different absolute amount of radio resources depending on the configured carrier bandwidth.

**Table 1:** Configured channel bandwidths of LTE and NR cells included in the dataset.

Technology	Band	Channel bandwidth
LTE	B20, B28	10 MHz
LTE	B1, B3	20 MHz
NR	n28	10 MHz
NR	n1, n3	20 MHz
NR	n78	100 MHz

### Data extraction

To process the large volume of XML files, a dedicated parser was developed to extract a predefined subset of network parameters and KPIs. The data extraction and transformation procedures were carried out in strict accordance with the official documentation of the mobile network operator to ensure accurate interpretation and consistent mapping of the raw performance data into a structured tabular dataset.

The base stations comprise multi-band radio units configured with multiple technologies (2G, 4G or 5G) within a single radio. Using the developed parser, PM counters were extracted separately for each configuration defined on every radio unit, corresponding to individual network cells. Each network cell is further divided into sectors, providing additional granularity, as PM counters are also extracted at the sector level. Consequently, the resulting dataset provides measurements at the cell and sector levels for each timestamp, with every cell characterised by its network technology and operating frequency.

### Anonymisation

The presented dataset is provided in anonymized form to protect sensitive and proprietary information. Identifiers and labels associated with base stations and sectors are replaced with anonymous codes. The anonymization preserves internal consistency, such that all records originating from the same base station and sector retain identical anonymous identifiers.

## Data Records

### Repository and access

The dataset presented in this paper^6^ is available for download from Zenodo (https://doi.org/10.5281/zenodo.17815388). The Zenodo record provides three independent ZIP archives (Dataset_01-Dataset_03), each corresponding to a distinct data collection period and subset of base stations (see Directory layout and Table 2). Each ZIP archive is self-contained and organised into per-technology folders, enabling users to download and work with any subset independently.

**Table 2:** Dataset partition key characteristics and basic distributions.

Subset	Network configurations	Observation days	Base stations	Records
Dataset_01	LTE 2100 MHz, NR 1800 MHz	35 days	25	302 052
Dataset_02	LTE 1800 MHz, NR 2100 MHz	64 days	63	3 601 840
Dataset_03	GSM 900 MHz, LTE 700/800/1800 MHz, NR 2100/3500 MHz	84 days	264	30 213 561

The dataset is released under the Creative Commons Attribution 4.0 International (CC BY 4.0) license. When citing or reusing the dataset, both this Data Descriptor and the Zenodo record^6^ should be cited. Where applicable, the specific version of the dataset used should be indicated.

### Directory layout

The dataset structure was designed to reflect a real-world base station infrastructure, encompassing multiple network technologies and frequency bands. The data is organised into three temporal partitions, each representing a distinct data collection period and containing different subsets of base stations. Within each temporal partition (*Dataset_01* to *Dataset_03*), data are further structured by basebands, which represent separate processing units within the base stations. Each baseband folder contains measurements collected from multiple network cells defined on multi-band radio units, corresponding to the physical transmission hardware deployed at the site. Network cells (radio configurations) specify the deployed mobile network technologies (e.g., GSM, LTE, NR) and their associated frequency bands. Each radio configuration is represented by a dedicated Comma-Separated Values (CSV) file containing the measurement data. The sector-level division is applied directly in CSV files, where each record corresponds to measurements from a specific sector of a radio unit.

The dataset partitions are distinguished by their respective network configurations, observation period and the number of base stations and measurement records. These characteristics are summarised in the Table 2.

The hierarchical organisation of the dataset is illustrated as follows:

To protect sensitive deployment information, base-station identifiers are provided in anonymized form using consistent pseudonyms. The internal relationships between anonymized base stations, basebands, radio units and sectors are preserved, maintaining structural integrity while preventing direct mapping of the anonymized identifiers to real network sites.

### Primary files

Table 3 summarises the primary dataset files and their respective contents.

**Table 3:** Overview of primary files.

File / folder	Description
Dataset_XX_*.csv	CSV files containing cellular measurement data for GSM, LTE, and NR technologies, with sector-level observations per network cell

### Field dictionary

The CSV files contain measurement records aggregated over 15-minute intervals. Each record includes three mandatory attributes that are consistent across all network technologies:

- **Base station** - anonymous identifier for the base station,
- **Sector** - sector number within the radio unit,
- **Timestamp** - measurement datetime in Coordinated Universal Time (UTC).

Beyond these attributes, CSV files contain technology-specific features that vary depending on the mobile network generation being measured (2G, 4G, or 5G). The dataset comprises key mobile network parameters, including Resource Block (RB)/Time Slots (TS) utilisation, Channel Quality Indicator (CQI), Radio Resource Control (RRC) connected users, active users, data volume, and MIMO rank, with the majority of parameters distinguished by uplink (UL) and downlink (DL) directions. These attributes are organized into three feature groups corresponding to each technology type. Each individual CSV file is dedicated to a specific technology/frequency configuration and contains only the feature group associated with that network generation. A detailed description of the technology-specific features is provided in Table 4.

**Table 4:** Technology-specific parameters definitions and units.

Field	Units	Meaning/notes
4G RB utilization	%	Average resource block utilization
4G CQI rank 1	absolute number	Average reported CQI for rank 1 transmissions
4G CQI rank 2	absolute number	Average reported CQI for rank 2 transmissions
4G CQI rank 3	absolute number	Average reported CQI for rank 3 transmissions
4G CQI rank 4	absolute number	Average reported CQI for rank 4 transmissions
4G RRC users	absolute number	Average number of RRC connected users
4G max RRC users	absolute number	Maximum number of RRC connected users
4G active users UL	absolute number	Average number of active users in UL
4G active users DL	absolute number	Average number of active users in DL
4G max active users UL	absolute number	Maximum number of active users in UL
4G max active users DL	absolute number	Maximum number of active users in DL
4G data volume UL	megabytes	Total transmitted data volume in UL
4G data volume DL	megabytes	Total transmitted data volume in DL
4G MIMO rank DL	absolute number	Average used transmission rank in DL
5G RB utilization	%	Average resource block utilization
5G CQI rank 1	absolute number	Average reported CQI for rank 1 transmissions
5G CQI rank 2	absolute number	Average reported CQI for rank 2 transmissions
5G CQI rank 3	absolute number	Average reported CQI for rank 3 transmissions
5G CQI rank 4	absolute number	Average reported CQI for rank 4 transmissions
5G RRC users	absolute number	Average number of RRC connected users
5G max RRC users	absolute number	Maximum number of RRC connected users
5G active users UL	absolute number	Average number of active users in UL
5G active users DL	absolute number	Average number of active users in DL
5G max active users UL	absolute number	Maximum number of active users in UL
5G max active users DL	absolute number	Maximum number of active users in DL
5G data volume UL	megabytes	Total transmitted data volume in UL
5G data volume DL	megabytes	Total transmitted data volume in DL
5G MIMO rank DL	absolute number	Average used transmission rank in DL
2G TS available	absolute number	Available time slots
2G TS used	absolute number	Time slots used
2G TS utilization	%	Time slots utilization
Radio unit energy consumption	watt-hours	Total energy consumption at the radio unit level over a 15-minute interval
Baseband energy consumption	watt-hours	Total energy consumption at the baseband level over a 15-minute interval

RB utilisation values should be interpreted together with the configured channel bandwidths listed in Table 1, because the same percentage utilisation corresponds to a different absolute amount of available radio resources depending on the carrier bandwidth.

For the CQI field, the dataset contains CQI index values reported by user equipment (UE) and recorded in standard RAN performance management (PM) statistics. These values follow the CQI index definitions in 3GPP TS 36.213 for LTE and 3GPP TS 38.214 for NR^7,8^. However, the dataset does not include the specific CQI table configuration used in the network, such as tables associated with up to 64QAM or 256QAM. Because the mapping from CQI index to modulation order, code rate, and spectral efficiency depends on the configured CQI table (which may vary across cells, frequency bands, and time), CQI values in this dataset should therefore be interpreted as relative indicators of downlink channel quality as reported by UEs, rather than as uniquely defined physical layer quantities. Analyses requiring absolute physical layer interpretation must therefore make explicit assumptions about the CQI table or rely on external calibration. This is consistent with standard field-collected RAN PM data, where CQI indices are typically recorded without explicit linkage to the active CQI table.

Energy consumption is reported as the total electrical energy consumed during each 15-minute interval, measured in watt-hours (Wh). An important characteristic of this dataset is the granularity of energy consumption measurements. Energy consumption is measured and reported at the radio unit and sector level rather than being attributed to individual network cells. As a result, when multiple network cells, each representing different network technology and frequency combination, operate on the same radio unit and sector, they share the energy consumption value. This shared energy attribution has important implications for data analysis. First, cell-level performance assessments must account for the absence of direct, cell-specific energy consumption metrics. Second, when merging or joining datasets from different network cells that belong to the same baseband and sector, careful attention must be paid to avoid duplicating energy consumption values, as the same measurement applies to all cells sharing that radio resource.

## Technical Validation

### Data validation and quality control

The dataset originates from a commercial cellular network where data collection, counter aggregation and exclusion rules are performed by the mobile network operator. Therefore, no synthetic records were generated and no post-processing was applied that altered the original operator-defined measurement semantics.

To ensure the dataset is technically correct and usable for research, several data validation steps were performed prior to publication. All records were checked for the presence of mandatory attributes (*Base station*, *Sector*, *Timestamp*) and technology-specific features. Across all datasets, about 17% of records contain at least one missing KPI value. The majority of missing entries occur in utilisation metrics, user counts and CQI reports. Rather than removing incomplete records, all missing or unavailable measurements were preserved in their original state. This approach reflects real-world data volatility (e.g. temporary outages, counter resets and maintenance windows) and allows researchers to apply data-driven methods to handle such events according to their specific analytical needs.

All records were checked for duplicates using the tuple (Base station, Sector, Timestamp). No duplicate rows were found, therefore no deduplication step was required. All attributes were validated for correct data types and format consistency throughout the dataset. Data format consistency checks included verification of correct data types (numeric, string, datetime), valid timestamp formatting, uniform decimal separators, and consistent column structures across all CSV files. Selected numerical features were examined to confirm values fall within expected realistic ranges which are presented in Table 5.

**Table 5:** Expected KPI ranges.

KPI	Expected range	Rationale
Energy consumption (Wh)	≥0	Negative consumption is physically impossible
RB utilisation (%)	0–100	Represents the fraction of allocated resource blocks
Active users	≥0	User-count metrics are non-negative values
CQI (4G/5G)	0–15	Defined by 3GPP CQI index range
MIMO rank (4G/5G)	1–4	Depends on supported transmission modes

**Fig. 2:**
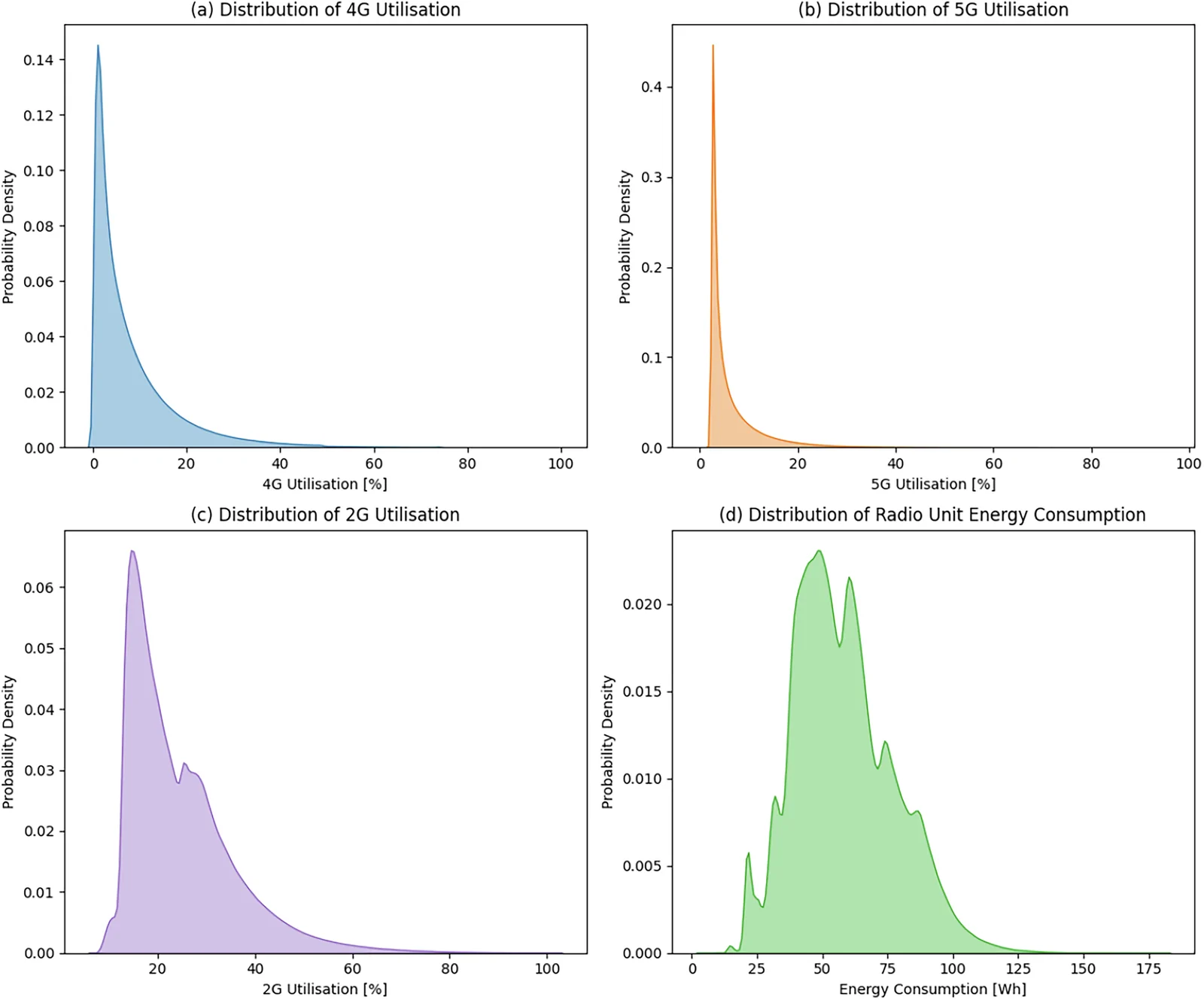
Probability distributions of (**a**) 4G utilisation, (**b**) 5G utilisation, (**c**) 2G utilisation and (**d**) radio unit energy consumption across the collected dataset.

Figure 2 presents the probability density distributions of network utilisation across 5G, 4G, and 2G technologies, along with radio unit energy consumption. Both 4G and 5G networks operate predominantly at low utilisation levels, with 5G utilisation being even more constrained than 4G. This pattern is consistent with real-world network conditions during the collection period, where 5G infrastructure was less intensively used, likely due to lower subscriber and device adoption as well as deployment-specific coverage.

### Handling invalid values

#### Energy consumption

Missing values in energy consumption metrics typically indicate network outages or measurement system failures. Under normal operation, both radio and baseband units consume a non-negligible amount of power even during intervals with no user traffic. Therefore, zero values in radio unit or baseband energy consumption should not be interpreted as valid zero-consumption operational states; they indicate that the corresponding unit did not report energy consumption correctly during the given 15-minute interval or that the unit was physically turned off. In contrast, zero values in traffic-related KPIs may represent valid low-traffic or no-traffic intervals. However, records where energy consumption metrics and all or nearly all other KPI values are simultaneously missing or zero should be treated as unavailable or non-operational intervals, rather than as normal no-traffic states.

#### Utilisation metrics

Missing values for 2G/4G/5G utilisation metrics are generally caused by zero or extremely low load. These can be safely imputed with zero, as they represent periods of minimal network traffic.

#### User counts

Missing values for RRC connected and active users typically occur during zero or low traffic periods and can be replaced with zero to accurately reflect the absence of connected devices.

#### CQI and MIMO rank

For CQI and MIMO rank, we identified missing or zero values that occur when no devices send reports during zero-traffic intervals. For analysis purposes, these zeros can be imputed with temporal averages from the same base station and sector within a relevant time window to maintain realistic operational patterns.

### Exploratory data analysis

To verify technical plausibility, Exploratory Data Analysis (EDA) was performed using various visualization techniques including scatter plots, boxplots, correlation matrices, and distribution histograms. Through these visualizations, we validated that the network behavior aligns with established mobile network theory and that the data exhibits expected operational patterns.

### Outlier inspection and validation

During EDA, we identified a number of measurements that deviate significantly from the dominant operational behaviour of individual base stations. In this context, an outlier refers to a value that falls outside the typical operational range for a given KPI, yet may still occur naturally in commercial mobile networks.

These outliers were intentionally preserved rather than removed, as they represent realistic network phenomena. Commercial RAN deployments routinely exhibit short-term irregularities caused by maintenance operations, counter resets, sudden bursts of user activity, or temporary hardware issues. Such events create anomalous KPI patterns that are valuable for both operational diagnostics and research use cases.

These deviations are therefore meaningful rather than artefacts of data corruption. Preserving them ensures that the dataset remains suitable for anomaly detection studies, traffic irregularity modelling and validating algorithms under realistic operational conditions.

## Data Availability

The anonymized cellular base-station counter dataset described in this Data Descriptor is openly available in the Zenodo repository at https://doi.org/10.5281/zenodo.17815388^6^ under a CC BY 4.0 licence. The repository contains all data partitions (files) required to reuse the dataset.

## Code availability

Custom scripts were used to extract and convert measurement files provided by a mobile network operator from a proprietary vendor-specific format into the released dataset. These scripts are not publicly available because they are tied to proprietary internal systems and their release is not permitted. No custom code is required to access or reuse the dataset published in the Zenodo repository. The released files are provided in directly usable form as described in this Data Descriptor.

## References

[1] Almeida, A., Rito, P., Brás, S., Pinto, F. C. & Sargento, S. A machine learning approach to forecast 5G metrics in a commercial and operational 5G platform: 5G and mobility. *Computer Communications* **228**, 10.1016/j.comcom.2024.107974 (2024).

[2] Eller, L., Svoboda, P. & Rupp, M. A Differentiable Throughput Model for Load-Aware Cellular Network Optimization Through Gradient Descent. *IEEE Access* 10.1109/ACCESS.2024.3356049 (2024).

[3] Kousias, K., Vlachou, C., Bouras, C. & Kioumourtzis, G. A Public Multi-Technology Dataset of Mobile Network Measurements for NB-IoT, LTE and 5G NSA. *Sensors* **23**, 10.3390/s23073611 (2023).

[4] Schippers, T., Simsek, M., Sayrac, B., Amirijoo, M. & Kellerer, W. DoNext: An Open Dataset for 4G/5G Mobile Network Measurement Analysis. *Proceedings of ACM CoNEXT* 10.1145/3623741.3624493 (2023).

[5] Chai, H., Zhang, S., Qi, X., Qiu, B. & Li, Y. UoMo: a universal model of mobile traffic forecasting for wireless network optimization. In *Proceedings of the 31st ACM SIGKDD Conference on Knowledge Discovery and Data Mining, KDD* ’25, 4308–4319, ACM 10.1145/3711896.3737272 (2025).

[6] Lehoczký, P. *et al*. Performance Management Counters from Live 5G, 4G and 2G Radio Access Network [Dataset]. *Zenodo*. 10.5281/zenodo.17815388 (2026). PMC13696700 42399262

[7] 3GPP. *Evolved Universal Terrestrial Radio Access (E-UTRA); Physical layer procedures*. 3GPP TS 36.213, version 16.11.0, Release 16 (2025).

[8] 3GPP. *NR; Physical layer procedures for data*. 3GPP TS 38.214, version 17.11.0, Release 17 (2024).

